# Association between cumulative intraoperative hypotension and postoperative ICU admission after gastrointestinal lumen cancer surgery: a retrospective cohort study using the INSPIRE database

**DOI:** 10.3389/fmed.2026.1916589

**Published:** 2026-08-19

**Authors:** Zhendong Liu, Ning Wang, Min Chen, Hongyan Wang

**Affiliations:** 1 School of Nursing, Gansu University of Chinese Medicine, Lanzhou, Gansu, China; 2 Gansu Provincial Maternal and Child Health Care Hospital, Lanzhou, Gansu, China

**Keywords:** gastrointestinal lumen cancer surgery, INSPIRE database, intensive care unit admission, intraoperative hypotension, mean arterial pressure, perioperative medicine

## Abstract

**Background:**

Intraoperative hypotension is associated with postoperative organ injury, but its relationship with postoperative intensive care unit (ICU) admission after gastrointestinal lumen cancer surgery remains uncertain.

**Methods:**

We conducted a retrospective cohort study using the INSPIRE perioperative database. Adults undergoing their first gastrointestinal lumen excision or resection, identified by ICD-10-PCS 0DB*/0DT* codes and a temporally aligned ICD-10-CM C15–C21 diagnosis, were included. The primary exposure was cumulative duration of mean arterial pressure (MAP) <65 mmHg, categorized as 0, >0 to <10, 10 to <30, and ≥30 min. Multivariable logistic regression adjusted for demographic, clinical, laboratory, and surgical factors. Secondary analyses characterized the empirical MAP measurement intervals, time-weighted area below MAP 65 mmHg, and hypotension burden normalized to the monitored, strict operation, and strict anesthesia windows.

**Results:**

Among 7,405 patients, 1,157 (15.62%) were admitted to the ICU. ICU admission rates were 7.15, 10.91, 15.25, and 32.01% across increasing exposure categories. In the complete-case expanded model (*n* = 6,045; 966 events), adjusted odds ratios were 1.02 (95% CI, 0.75–1.40), 1.29 (95% CI, 1.00–1.66), and 1.78 (95% CI, 1.36–2.33), respectively, compared with no exposure. Of 319,661 consecutive MAP intervals, 99.43% were 5 min. Each 50-mmHg·min increase in the area below MAP 65 mmHg was associated with an OR of 1.07 (95% CI, 1.04–1.10), and each 5-min/h increase in MAP <65 mmHg duration within the strict operation window with an OR of 1.14 (95% CI, 1.07–1.21); both *p* < 0.001.

**Conclusion:**

Sustained intraoperative MAP <65 mmHg, particularly for ≥30 min, was associated with higher odds of postoperative ICU admission after adjustment for measured clinical and surgical factors. Findings remained consistent when hypotension depth and time-normalized burden were considered, but do not establish causality or an intervention threshold.

## Introduction

Gastrointestinal cancers impose a substantial global health burden. Recent global estimates indicate that digestive system cancers account for nearly one quarter of all newly diagnosed cancers, with colorectal and gastric cancers among the most common malignancies worldwide (1, 2). Gastrointestinal lumen cancers, including malignancies of the esophagus, stomach, small intestine, colon, rectum, and anus, frequently require surgical excision or resection as a central component of curative-intent treatment. These procedures are often physiologically demanding and are commonly performed in older patients with reduced functional reserve, nutritional impairment, and coexisting illness (3, 4). Identifying perioperative factors associated with postoperative care requirements is therefore clinically important in this population.

Intraoperative hypotension (IOH) is common during noncardiac surgery and has been regarded as a potentially modifiable perioperative exposure. Its reported incidence varies according to the blood-pressure threshold, duration criterion, and monitoring frequency used (5, 6). Observational studies have consistently suggested that both the severity and cumulative duration of IOH are associated with postoperative organ injury. Postoperative delirium is another clinically important complication in vulnerable surgical patients (7). Salmasi and colleagues identified MAP <65 mmHg as a clinically relevant absolute threshold associated with acute kidney injury and myocardial injury after noncardiac surgery, with progressively greater risk at higher hypotension burdens (8). More recent work has also operationalized MAP <65 mmHg as a target for real-time prediction of intraoperative hypotension (9). Subsequent systematic reviews and meta-analyses have linked IOH with acute kidney injury, myocardial injury, stroke, and mortality (10–12). The Perioperative Quality Initiative consensus statement recognizes the range of approximately 60–70 mmHg as clinically important when evaluating and treating intraoperative blood-pressure reductions (13).

Important uncertainties nevertheless remain. Most previous investigations have examined heterogeneous noncardiac surgical populations or organ-specific outcomes. Perioperative risk-stratification studies have addressed postoperative hypotension and critical-care needs in other clinical settings (14–16), but postoperative intensive care unit (ICU) admission has received less attention as an outcome in gastrointestinal cancer surgery. ICU admission is clinically and operationally important because it represents substantial postoperative monitoring and resource use. However, it is not equivalent to a postoperative complication: it may reflect unplanned escalation of care, anticipated monitoring after complex surgery, preoperative triage decisions, or institutional ICU practice. Accordingly, the relationship between IOH and postoperative ICU admission should be interpreted as an association with postoperative care requirements rather than direct evidence of hypotension-related deterioration.

Patients undergoing gastrointestinal lumen cancer surgery may be particularly susceptible to the consequences of reduced perfusion pressure because anemia, hypoalbuminemia, malnutrition, cancer-related inflammation, bowel preparation, major abdominal or thoracic exposure, and perioperative fluid shifts may limit physiologic reserve (17). Recent prognostic modeling studies in gastric cancer further underscore the heterogeneity of postoperative risk in this population (18). At the same time, surgical duration and complexity may influence both cumulative hypotension burden and the decision to admit a patient to the ICU. Analyses of this relationship therefore require careful adjustment for measured clinical risk factors as well as available surgical characteristics. The extent to which cumulative IOH remains associated with postoperative ICU admission after accounting for these factors has not been well characterized in patients undergoing gastrointestinal lumen cancer surgery (19).

The INSPIRE database provides an opportunity to examine this question. INSPIRE is a perioperative research database derived from surgical care at Seoul National University Hospital and includes time-series intraoperative vital signs, laboratory measurements, perioperative variables, diagnoses, procedures, and postoperative outcomes (20). Its dense intraoperative MAP recordings permit reconstruction of cumulative hypotension duration below clinically meaningful thresholds rather than classification based only on the occurrence of a single low value. The available procedural and clinical data also support adjustment for operation duration, procedure site, surgical approach, anesthesia type, and preoperative physiologic characteristics, together with sensitivity analyses using alternative exposure and cohort definitions.

In this study, we evaluated the association between intraoperative hypotension burden and postoperative ICU admission among adults undergoing gastrointestinal lumen cancer excision or resection. The primary exposure was cumulative duration of MAP <65 mmHg, and the primary outcome was postoperative ICU admission during the index hospitalization. We hypothesized that longer cumulative MAP <65 mmHg exposure would be associated with higher odds of ICU admission after adjustment for measured clinical and surgical factors. Secondary objectives were to characterize the dose–response relationship, quantify the time-weighted area below MAP thresholds as an integrated measure of hypotension depth and duration, normalize hypotension burden to the observed monitoring, operation, and anesthesia windows, evaluate robustness across alternative cohort and missing-data analyses, and assess potential effect modification across clinically relevant subgroups.

## Methods

### Study design and data source

We conducted a retrospective cohort study using the INSPIRE (Integrated Study of Perioperative Medicine in Real-world Evidence) database, a publicly available perioperative dataset containing de-identified surgical, anesthesia, time-series vital-sign, laboratory, medication, diagnosis, and postoperative outcome data from Seoul National University Hospital (20–22). The study was reported in accordance with the Strengthening the Reporting of Observational Studies in Epidemiology (STROBE) recommendations. The creation and release of INSPIRE were approved by the Institutional Review Board of Seoul National University Hospital, and the requirement for individual informed consent was waived because the data were fully de-identified. The present study was a secondary analysis of anonymized clinical data and involved no participant contact or clinical intervention.

### Study population and cohort reconstruction

All operations in INSPIRE were screened. Patients were eligible if they were aged 18 years or older and underwent a gastrointestinal lumen excision or resection procedure with a temporally aligned malignant neoplasm diagnosis corresponding to ICD-10-CM codes C15–C21. The INSPIRE procedure field contains shortened 5- to 7-character ICD-10-PCS strings that retain the section, body system, root operation, body part, and approach characters. For the primary strict cohort, eligible procedures began with 0DB (Excision) or 0DT (Resection) and involved a gastrointestinal lumen body part corresponding to the esophagus or esophagogastric junction, stomach, small intestine or appendix, colon, rectum, anus, or anal canal. Procedures limited to the omentum, mesentery, or peritoneum were excluded. Inspection, drainage, repair, insertion, and other non-excisional procedures were not included in the primary cohort.

A C15–C21 diagnosis was considered temporally aligned when it was recorded during the same hospital admission, allowing a 1-day timestamp buffer, or within 30 days before or after the index operation. Procedure and diagnosis criteria were applied before selecting the first eligible operation for each patient. Elective and emergency procedures, open and minimally invasive approaches, and general and non-general anesthesia were eligible. Operations were classified according to the recorded primary procedure code; because the available procedure field did not provide a complete set of concurrent secondary codes, combined procedures could not be comprehensively identified and were not used as an exclusion criterion. Patients were excluded if the postoperative ICU admission indicator was missing or if fewer than two distinct physiologically valid intraoperative MAP time points were available.

Two alternative cohort definitions were evaluated. A broad gastrointestinal procedure cohort included any gastrointestinal lumen procedure within the 0D body system with a temporally aligned C15–C21 diagnosis, irrespective of the recorded root operation. A site-compatible strict cohort additionally required anatomical compatibility between the procedure body part and the aligned cancer diagnosis. A further sensitivity analysis excluded esophageal procedures and operations performed by the cardiothoracic surgery service because postoperative ICU admission was nearly routine in this subgroup.

### Intraoperative hypotension exposure

Intraoperative MAP measurements were reconstructed from invasive arterial MAP and noninvasive blood-pressure MAP records in the raw vital-sign table. When both sources were recorded at the same timestamp, invasive arterial MAP was preferentially selected. MAP values outside the prespecified physiologically plausible range of 20–250 mmHg were removed. For each operation, valid MAP measurements were ordered chronologically, and the empirical distribution of consecutive measurement intervals was summarized. Each measurement was treated as representing the interval until the next valid measurement, with the represented interval capped at 5 min to prevent prolonged recording gaps from inflating hypotension duration or time-weighted burden. The observed MAP interval was defined as the sum of these capped consecutive intervals.

The primary exposure was cumulative duration of MAP <65 mmHg, categorized as 0 min, >0 to <10 min, 10 to <30 min, and ≥30 min. Alternative threshold and severity measures were MAP <65 mmHg for at least 10 min, MAP <60 mmHg for at least 5 min, MAP <65 duration per 10-min increment, MAP <60 duration per 5-min increment, and the lowest intraoperative MAP per 5-mmHg decrease from 80 mmHg. To integrate hypotension depth and duration, the time-weighted area below MAP 65 mmHg was calculated as the sum of (65 − MAP) multiplied by the represented interval for values below 65 mmHg; the corresponding area below MAP 60 mmHg was calculated analogously. Areas were expressed in mmHg·min.

To account for differences in the opportunity to accumulate hypotension, burden was normalized to time. For the whole monitoring window, we calculated the percentage of observed MAP time below 65 mmHg and minutes below 65 mmHg per monitored hour. We also constructed strict operation and anesthesia windows using the recorded operation-start/operation-end and anesthesia-start/anesthesia-end timestamps. Each represented MAP interval was clipped to its overlap with the relevant window before calculating duration and area; the resulting measures were expressed per operation or anesthesia hour. MAP coverage was calculated as the represented MAP time within each strict window divided by the corresponding window duration.

### Outcome

The primary outcome was postoperative ICU admission during the index hospitalization, defined using the binary ICU admission indicator provided in INSPIRE. The database did not contain a validated field distinguishing planned from unplanned ICU admission; therefore, the outcome was interpreted as a combined marker of postoperative care escalation, monitoring requirements, resource use, surgical complexity, and institutional practice rather than as a postoperative complication alone.

### Covariates

Clinical covariates were selected on the basis of prior perioperative evidence, clinical plausibility, and availability in INSPIRE. They included age, sex, American Society of Anesthesiologists (ASA) physical status, emergency operation status, body mass index (BMI), hemoglobin, albumin, creatinine, and glucose. ASA class was grouped as 1, 2, and ≥3. Surgical variables available for expanded confounding adjustment included operation duration, procedure site, surgical approach, and anesthesia type. Procedure site was classified as colorectal/anal, gastric, small bowel/appendix, esophageal/esophagogastric junction, or other/unspecified. Surgical approach was classified as open, minimally invasive, or other, and anesthesia type as general or other. An alternative expanded model used cancer site derived from the aligned C15–C21 diagnosis instead of procedure site.

Cancer stage, planned ICU status, and consistently linkable operation-level measures of blood loss, transfusion, fluid administration, and vasopressor dose were not available in a sufficiently complete and validated form for the full cohort. Vasopressor and fluid administration may also occur in response to hypotension and were therefore not considered baseline confounders. ASA class and the available preoperative laboratory variables were used to capture overall preoperative risk and physiologic reserve.

### Statistical analysis

Continuous variables are presented as medians with interquartile ranges (IQRs), and categorical variables as counts and percentages. Baseline characteristics were summarized across the four MAP <65 mmHg duration categories. Overall between-group differences were evaluated using the Kruskal–Wallis test for continuous variables and the chi-square test for categorical variables. Trends across ordered exposure categories were evaluated using the exposure category as an ordinal score.

Logistic regression was used to estimate odds ratios (ORs) and 95% confidence intervals (CIs) for postoperative ICU admission. The crude model included only the exposure. Model 1 adjusted for age and sex. Model 2 additionally adjusted for ASA class and emergency operation. Model 3 additionally adjusted for BMI, hemoglobin, albumin, creatinine, and glucose and represented the prespecified clinical adjustment model. Model 4 further adjusted for the observed MAP interval and was treated as a sensitivity model. Model 5A added operation duration, procedure site, surgical approach, and anesthesia type to the Model 3 covariates and served as the principal expanded confounding analysis. Model 5B replaced procedure site with cancer site. Observed MAP interval and operation duration were evaluated in separate models because they represent closely related measures of procedural and monitoring time and were highly correlated. Multicollinearity in the expanded models was assessed using variance inflation factors (VIFs) calculated from treatment-coded design matrices with an intercept.

Dose–response relationships were examined by modeling the ordered exposure category as a linear score and by fitting restricted cubic splines with 4 degrees of freedom. Likelihood-ratio tests compared reduced, linear, and spline models to evaluate the overall association and departure from linearity. Alternative threshold and severity definitions were analyzed using Models 3, 4, and 5A. Time-weighted and normalized metrics were evaluated in Model 5A using clinically interpretable increments: 50 mmHg·min for the whole-window area below MAP 65 mmHg, 5 percentage points for the proportion of monitored time below 65 mmHg, 5 min/h for normalized hypotension duration, and 25 mmHg·min/h for normalized area. Restricted cubic spline analyses were repeated for strict operation-window duration and area. To assess sensitivity to incomplete intraoperative coverage, strict operation-window analyses were repeated among operations with MAP coverage ≥80% and ≥90%.

Subgroup analyses used the binary exposure of MAP <65 mmHg for at least 10 min. Prespecified subgroups were age (<65 vs. ≥65 years), sex, ASA class (1–2 vs. ≥3), emergency status, availability of invasive arterial MAP, operation duration (<median vs. ≥median), procedure site, and esophageal/cardiothoracic surgery status. Subgroup models used the Model 3 covariate set, excluding the stratification variable where applicable. Interaction *p* values were obtained from likelihood-ratio tests comparing models with and without exposure-by-subgroup interaction terms.

The primary regression analyses used complete cases. Missing-data patterns and differences between included and excluded patients were described. As a sensitivity analysis, missing BMI, hemoglobin, albumin, creatinine, and glucose values were handled using multiple imputation by chained equations with Bayesian ridge regression, posterior sampling, and 20 imputed datasets. The imputation model included the outcome, hypotension exposure, all model covariates, MAP metrics, operation duration, observed MAP interval, procedure site, approach, anesthesia type, and cancer site. Estimates were combined using Rubin’s rules. Additional sensitivity analyses used the broad procedure cohort, the site-compatible strict cohort, excluded esophageal/cardiothoracic operations, and substituted cancer site for procedure site. All tests were two-sided, and *p* < 0.05 was considered statistically significant. Analyses were performed in Python using pandas, NumPy, SciPy, statsmodels, patsy, scikit-learn, and matplotlib.

## Results

### Patient selection and analytic cohort

**Figure 1:**
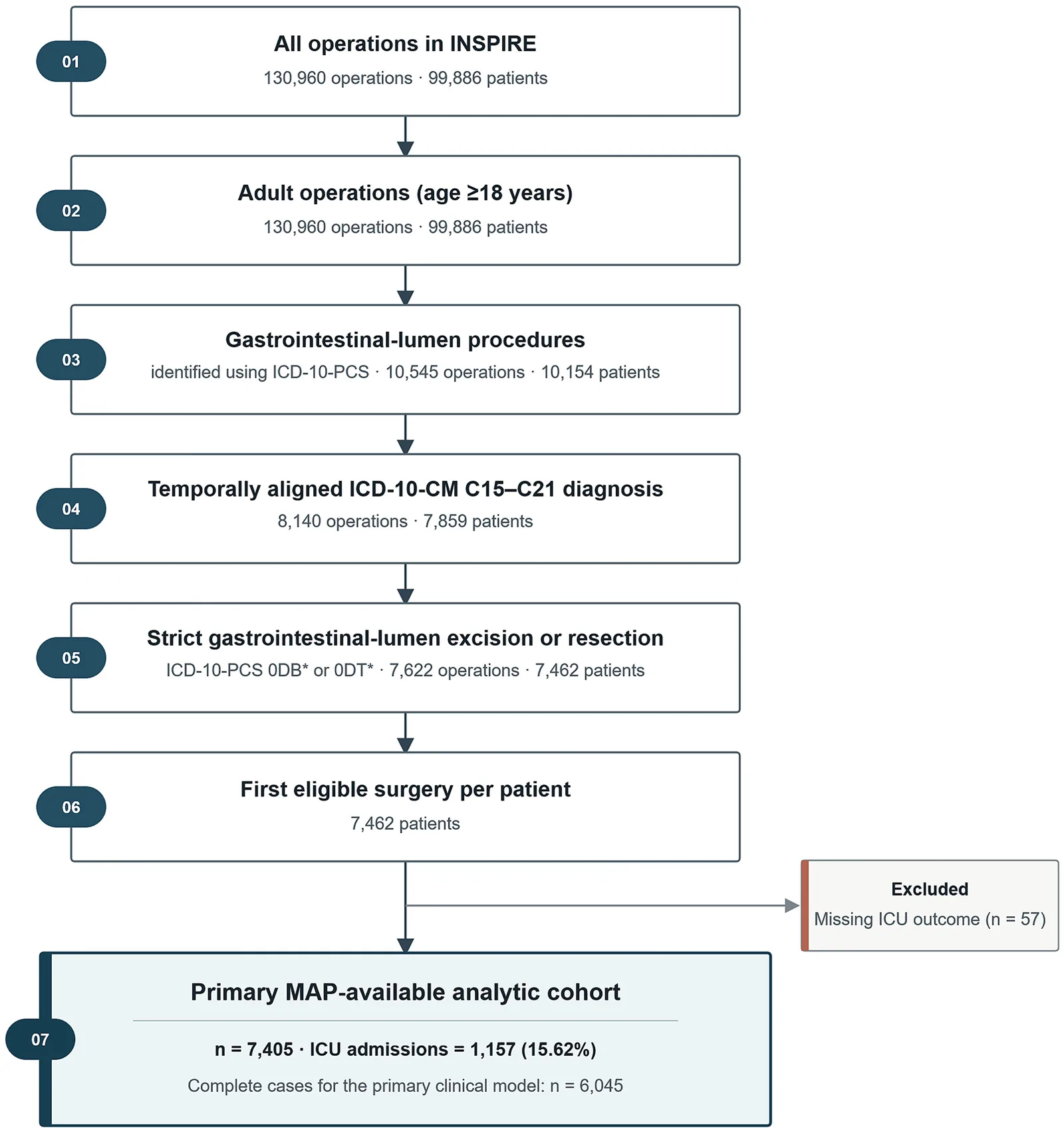
Flow diagram of study cohort selection. Eligible gastrointestinal lumen cancer excision or resection procedures were identified using ICD-10-PCS 0DB*/0DT* codes and temporally aligned ICD-10-CM C15–C21 diagnoses. The final analytic cohort included 7,405 patients. ICU, intensive care unit; MAP, mean arterial pressure.

Among 130,960 adult operations in 99,886 patients, 10,545 operations had a gastrointestinal lumen procedure code. Of these, 8,140 had a temporally aligned C15–C21 cancer diagnosis and 7,622 met the strict Excision/Resection procedure definition. After retaining the first eligible operation per patient, 7,462 patients remained. Fifty-seven patients were excluded because postoperative ICU admission status was missing. All remaining patients had at least two distinct valid intraoperative MAP time points, yielding a primary analytic cohort of 7,405 patients (Figure 1). Postoperative ICU admission occurred in 1,157 patients (15.62%). The complete-case cohort for Model 3 and the expanded models included 6,045 patients, with 966 ICU admissions.

The median age was 65 years (IQR, 55–70), 4,644 patients (62.7%) were male, and 311 (4.2%) underwent emergency surgery. The median operation duration was 170 min (IQR, 105–225). Procedure sites were colorectal/anal in 3,777 patients (51.0%), gastric in 3,236 (43.7%), esophageal/esophagogastric junction in 334 (4.5%), small bowel/appendix in 46 (0.6%), and other/unspecified in 12 (0.2%). General anesthesia was used in 7,317 patients (98.8%). The median number of MAP records was 42 (IQR, 29–54), the median observed MAP interval was 205 min (IQR, 140–265), the median lowest MAP was 62.0 mmHg (IQR, 58.5–65.5), and the median cumulative duration of MAP <65 mmHg was 10 min (IQR, 0–25).

### MAP measurement intervals and window coverage

The 7,405 operations contributed 327,066 selected MAP records and 319,661 consecutive measurement intervals. The median interval was 5 min (IQR, 5–5), and 317,853 intervals (99.43%) were exactly 5 min; 1,418 (0.44%) were >5 to ≤10 min and 390 (0.12%) were >10 min. Because each represented interval was capped at 5 min, longer recording gaps did not contribute more than 5 min to duration or area calculations. Strict operation windows were valid for all patients, with a median MAP coverage of 100% (IQR, 98.85–100%); 98.76 and 95.76% of operations had coverage ≥80% and ≥90%, respectively. Strict anesthesia windows were valid for 7,392 patients, with median coverage of 98.32% (IQR, 96.41–100%).

### Baseline characteristics by hypotension duration

Patients with longer cumulative MAP <65 mmHg duration were older and had lower BMI, hemoglobin, and albumin levels, higher ASA class, and longer operations and observed MAP intervals (all *p* for trend < 0.001 for these continuous variables). The proportion of esophageal/esophagogastric junction procedures increased from 1.2% in the 0-min group to 11.9% in the ≥30-min group. ICU admission rates increased progressively across the exposure categories: 7.15% (154/2,153) for 0 min, 10.91% (130/1,192) for >0 to <10 min, 15.25% (388/2,545) for 10 to <30 min, and 32.01% (485/1,515) for ≥30 min (*p* < 0.001; Table 1).

**Table 1:** Baseline characteristics according to cumulative MAP <65 mmHg duration.

Variable	Total	0 min	>0 to <10 min	10 to <30 min	≥30 min	*p* overall	*p* trend
Age, years	65.0 (55.0, 70.0)	60.0 (50.0, 65.0)	60.0 (55.0, 70.0)	65.0 (55.0, 70.0)	65.0 (55.0, 75.0)	<0.001	<0.001
BMI, kg/m^2^	22.89 (20.76, 25.39)	23.44 (21.22, 25.71)	23.15 (20.81, 25.39)	22.89 (20.76, 25.39)	22.49 (20.20, 24.97)	<0.001	<0.001
Hemoglobin, g/dL	12.90 (11.60, 14.20)	13.70 (12.50, 15.00)	13.20 (11.90, 14.20)	12.90 (11.30, 14.20)	12.50 (10.70, 13.70)	<0.001	<0.001
Albumin, g/dL	4.00 (3.80, 4.30)	4.10 (3.90, 4.30)	4.00 (3.90, 4.30)	4.00 (3.80, 4.20)	4.00 (3.70, 4.20)	<0.001	<0.001
Creatinine, mg/dL	0.84 (0.71, 0.98)	0.84 (0.71, 0.98)	0.80 (0.68, 0.92)	0.80 (0.68, 0.98)	0.84 (0.71, 0.98)	0.003	0.407
Glucose, mg/dL	101.00 (91.00, 120.00)	101.00 (91.00, 120.00)	101.00 (91.00, 115.00)	101.00 (91.00, 115.00)	105.00 (91.00, 126.00)	<0.001	0.110
Operation duration, min	170.0 (105.0, 225.0)	140.0 (85.0, 200.0)	155.0 (100.0, 210.0)	170.0 (115.0, 225.0)	215.0 (160.0, 280.0)	<0.001	<0.001
Observed MAP interval, min	205.0 (140.0, 265.0)	175.0 (115.0, 235.0)	190.0 (130.0, 245.0)	210.0 (150.0, 265.0)	255.0 (200.0, 325.0)	<0.001	<0.001
Lowest MAP, mmHg	62.0 (58.5, 65.5)	68.5 (65.5, 73.0)	62.0 (58.5, 62.0)	58.5 (58.5, 62.0)	52.0 (52.0, 58.5)	<0.001	<0.001
MAP <65 duration, min	10.0 (0.0, 25.0)	0.0 (0.0, 0.0)	5.0 (5.0, 5.0)	15.0 (10.0, 20.0)	45.0 (35.0, 65.0)	<0.001	<0.001
MAP <60 duration, min	0.0 (0.0, 5.0)	0.0 (0.0, 0.0)	0.0 (0.0, 5.0)	5.0 (0.0, 5.0)	10.0 (5.0, 20.0)	<0.001	<0.001
Sex, *n* (%)							
Female	2,761 (37.3%)	744 (34.6%)	484 (40.6%)	1,021 (40.1%)	512 (33.8%)	<0.001	0.588
Male	4,644 (62.7%)	1,409 (65.4%)	708 (59.4%)	1,524 (59.9%)	1,003 (66.2%)		
ASA class, *n* (%)							
1	2,353 (31.8%)	835 (38.8%)	402 (33.7%)	760 (29.9%)	356 (23.5%)	<0.001	
2	4,572 (61.7%)	1,229 (57.1%)	726 (60.9%)	1,623 (63.8%)	994 (65.6%)		
≥3	480 (6.5%)	89 (4.1%)	64 (5.4%)	162 (6.4%)	165 (10.9%)		
Emergency operation, *n* (%)							
No	7,094 (95.8%)	2059 (95.6%)	1,155 (96.9%)	2,437 (95.8%)	1,443 (95.2%)	0.182	0.457
Yes	311 (4.2%)	94 (4.4%)	37 (3.1%)	108 (4.2%)	72 (4.8%)		
Procedure site, *n* (%)							
Colorectal/anal	3,777 (51.0%)	1,252 (58.2%)	636 (53.4%)	1,259 (49.5%)	630 (41.6%)	<0.001	
Gastric	3,236 (43.7%)	853 (39.6%)	516 (43.3%)	1,176 (46.2%)	691 (45.6%)		
Small bowel/appendix	46 (0.6%)	23 (1.1%)	5 (0.4%)	10 (0.4%)	8 (0.5%)		
Esophageal/EGJ	334 (4.5%)	25 (1.2%)	33 (2.8%)	96 (3.8%)	180 (11.9%)		
Other/unspecified	12 (0.2%)	0 (0.0%)	2 (0.2%)	4 (0.2%)	6 (0.4%)		
Surgical approach, *n* (%)							
Open	3,733 (50.4%)	1,090 (50.6%)	585 (49.1%)	1,250 (49.1%)	808 (53.3%)	<0.001	
Minimally invasive	3,651 (49.3%)	1,049 (48.7%)	605 (50.8%)	1,291 (50.7%)	706 (46.6%)		
Other	21 (0.3%)	14 (0.7%)	2 (0.2%)	4 (0.2%)	1 (0.1%)		
Anesthesia type, *n* (%)							
General	7,317 (98.8%)	2072 (96.2%)	1,188 (99.7%)	2,542 (99.9%)	1,515 (100.0%)	<0.001	<0.001
Other	88 (1.2%)	81 (3.8%)	4 (0.3%)	3 (0.1%)	0 (0.0%)		
Postoperative ICU admission, *n* (%)							
No	6,248 (84.4%)	1999 (92.8%)	1,062 (89.1%)	2,157 (84.8%)	1,030 (68.0%)	<0.001	<0.001
Yes	1,157 (15.6%)	154 (7.2%)	130 (10.9%)	388 (15.2%)	485 (32.0%)		

Values are median (IQR) or *n* (%). *p* overall was calculated using nonparametric tests for continuous variables and chi-square tests for categorical variables. *p* for trend was calculated across ordered MAP <65 mmHg duration categories where applicable. ASA, American Society of Anesthesiologists; BMI, body mass index; ICU, intensive care unit; MAP, mean arterial pressure; EGJ, esophagogastric junction.

### Association between MAP <65 mmHg duration and ICU admission

In unadjusted analyses, compared with no MAP <65 mmHg exposure, the ORs for postoperative ICU admission were 1.59 (95% CI, 1.24–2.03) for >0 to <10 min, 2.33 (95% CI, 1.92–2.84) for 10 to <30 min, and 6.11 (95% CI, 5.02–7.44) for ≥30 min. In the prespecified clinical Model 3, the corresponding adjusted ORs were 1.43 (95% CI, 1.07–1.91; *p* = 0.015), 2.05 (95% CI, 1.63–2.59; *p* < 0.001), and 4.88 (95% CI, 3.85–6.17; *p* < 0.001), respectively.

Adjustment for procedural or monitoring time substantially attenuated the estimates. In Model 4, which additionally adjusted for observed MAP interval, the ORs were 1.12 (95% CI, 0.82–1.52; *p* = 0.466), 1.36 (95% CI, 1.06–1.74; *p* = 0.016), and 1.75 (95% CI, 1.35–2.28; *p* < 0.001). In the expanded Model 5A, which adjusted for operation duration, procedure site, surgical approach, and anesthesia type in addition to the clinical covariates, brief exposure of >0 to <10 min was not associated with ICU admission (OR, 1.02; 95% CI, 0.75–1.40; *p* = 0.880), whereas exposure lasting 10 to <30 min (OR, 1.29; 95% CI, 1.00–1.66; *p* = 0.047) and ≥30 min (OR, 1.78; 95% CI, 1.36–2.33; *p* < 0.001) remained associated with higher odds of ICU admission (Table 2). Replacing procedure site with cancer site in Model 5B produced similar estimates: OR 1.06 (95% CI, 0.77–1.45), 1.30 (95% CI, 1.01–1.68), and 1.79 (95% CI, 1.36–2.34), respectively. The maximum VIF was 1.82 in Model 5A and 1.90 in Model 5B, indicating no concerning multicollinearity.

**Table 2:** Association between cumulative MAP <65 mmHg duration and postoperative ICU admission.

Model	>0 to <10 min	10 to <30 min	≥30 min	*p* for trend
Crude	1.59 (1.24–2.03)	2.33 (1.92–2.84)	6.11 (5.02–7.44)	<0.001
Model 1	1.47 (1.14–1.89)	2.08 (1.70–2.55)	4.96 (4.05–6.07)	<0.001
Model 2	1.43 (1.11–1.84)	2.00 (1.63–2.45)	4.61 (3.75–5.67)	<0.001
Model 3	1.43 (1.07–1.91)	2.05 (1.63–2.59)	4.88 (3.85–6.17)	<0.001
Model 4	1.12 (0.82–1.52)	1.36 (1.06–1.74)	1.75 (1.35–2.28)	<0.001
Model 5A	1.02 (0.75–1.40)	1.29 (1.00–1.66)	1.78 (1.36–2.33)	<0.001

Values are odds ratios (95% confidence intervals), with 0 min as the reference category. Model 1 adjusted for age and sex. Model 2 additionally adjusted for ASA class and emergency operation. Model 3 additionally adjusted for BMI, hemoglobin, albumin, creatinine, and glucose. Model 4 additionally adjusted for observed MAP interval. Model 5A included the Model 3 covariates plus operation duration, procedure site, surgical approach, and anesthesia type; observed MAP interval was not included. ICU, intensive care unit; MAP, mean arterial pressure.

### Trend and dose–response analyses

**Figure 2:**
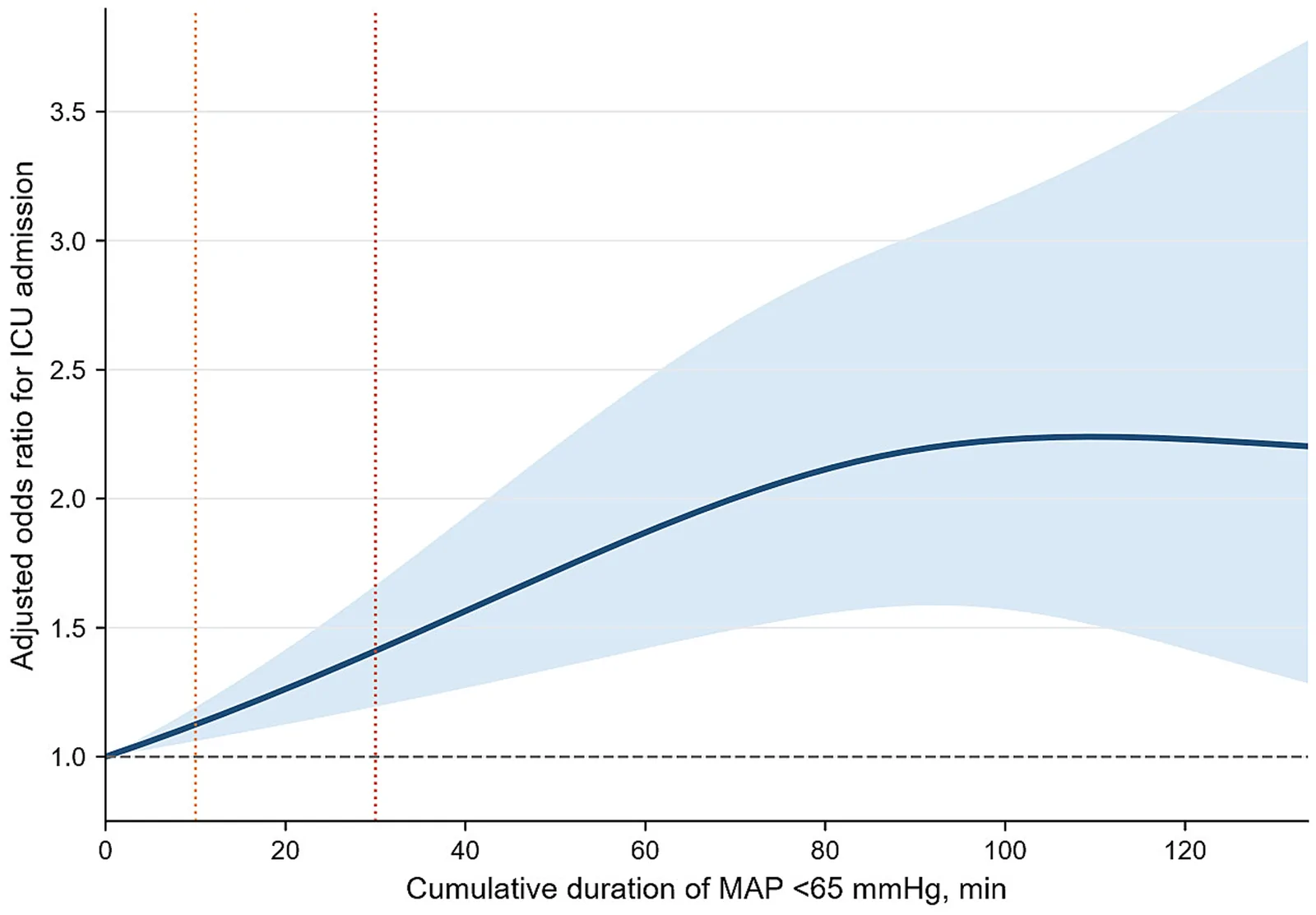
Cumulative intraoperative MAP <65 mmHg duration and postoperative ICU admission. The solid line shows adjusted odds ratios and the shaded area shows 95% confidence intervals. The restricted cubic spline model was adjusted for clinical and surgical factors, with 0 min as the reference. Overall association, *p* < 0.001; nonlinearity, *p* = 0.182. ICU, intensive care unit; MAP, mean arterial pressure.

The graded association remained significant across all sequential models. In Model 5A, each one-category increase in MAP <65 mmHg duration was associated with 22% higher odds of ICU admission (OR, 1.22; 95% CI, 1.12–1.33; *p* for trend < 0.001). Restricted cubic spline analysis also showed a significant overall association between continuous MAP <65 duration and ICU admission after adjustment for surgical factors (*p* overall < 0.001; Figure 2). Evidence of nonlinearity was present in Model 3 (*p* nonlinear = 0.001) but was attenuated after adjustment for observed MAP interval (*p* nonlinear = 0.319) or surgical factors (*p* nonlinear = 0.182), suggesting an approximately linear residual association after accounting for procedural duration and complexity.

### Alternative, time-weighted, and normalized exposure analyses

Results were consistent across alternative definitions of intraoperative hypotension. In Model 5A, MAP <65 mmHg for at least 10 min was associated with higher odds of ICU admission (OR, 1.44; 95% CI, 1.19–1.75; *p* < 0.001), as was MAP <60 mmHg for at least 5 min (OR, 1.23; 95% CI, 1.03–1.46; *p* = 0.023). Each additional 10 min of MAP <65 mmHg was associated with an OR of 1.08 (95% CI, 1.04–1.11; *p* < 0.001), each additional 5 min of MAP <60 mmHg with an OR of 1.10 (95% CI, 1.05–1.14; *p* < 0.001), and each 5-mmHg decrease in the lowest MAP from 80 mmHg with an OR of 1.16 (95% CI, 1.09–1.24; *p* < 0.001). The time-weighted area below MAP 65 mmHg was also associated with ICU admission (OR per 50 mmHg·min, 1.07; 95% CI, 1.04–1.10; *p* < 0.001) (Table 3).

**Table 3:** Alternative, time-weighted, and time-normalized definitions of intraoperative hypotension and postoperative ICU admission.

Exposure definition	Comparison or increment	Adjusted OR (95% CI)	*p* value
MAP <65 mmHg duration	≥10 min vs. <10 min	1.44 (1.19–1.75)	<0.001
MAP <60 mmHg duration	≥5 min vs. <5 min	1.23 (1.03–1.46)	0.023
Cumulative MAP <65 mmHg	Per 10-min increase	1.08 (1.04–1.11)	<0.001
Cumulative MAP <60 mmHg	Per 5-min increase	1.10 (1.05–1.14)	<0.001
Lowest intraoperative MAP	Per 5-mmHg decrease from 80 mmHg	1.16 (1.09–1.24)	<0.001
AUC below MAP 65 mmHg, whole monitoring window	Per 50-mmHg·min increase	1.07 (1.04–1.10)	<0.001
MAP <65 duration, whole monitoring window	Per 5-min/h increase	1.15 (1.08–1.23)	<0.001
MAP <65 duration, strict operation window	Per 5-min/h increase	1.14 (1.07–1.21)	<0.001
AUC below MAP 65 mmHg, strict operation window	Per 25-mmHg·min/h increase	1.11 (1.06–1.17)	<0.001

All estimates are from Model 5A, adjusted for age, sex, ASA class, emergency operation, BMI, hemoglobin, albumin, creatinine, glucose, operation duration, procedure site, surgical approach, and anesthesia type. The whole monitoring window comprised all represented MAP intervals. Strict operation-window metrics included only the overlap between represented MAP intervals and the recorded operation-start to operation-end window. AUC, area under the curve; ICU, intensive care unit; MAP, mean arterial pressure; OR, odds ratio.

Time-normalized analyses yielded consistent results. Each 5-min/h increase in MAP <65 mmHg duration across the whole monitoring window was associated with an OR of 1.15 (95% CI, 1.08–1.23; *p* < 0.001). When exposure was restricted to the interval between operation start and operation end, each 5-min/h increase in MAP <65 mmHg duration was associated with an OR of 1.14 (95% CI, 1.07–1.21; *p* < 0.001), and each 25-mmHg·min/h increase in the corresponding area was associated with an OR of 1.11 (95% CI, 1.06–1.17; *p* < 0.001). Strict anesthesia-window estimates were similar. Results remained consistent in operations with MAP coverage ≥80% or ≥90%. The strict operation-window spline analyses showed significant overall associations for normalized duration and area (both *p* < 0.001), without clear evidence of nonlinearity (*p* = 0.174 and *p* = 0.377, respectively).

### Sensitivity analyses

The expanded-model results were materially unchanged after excluding esophageal procedures and cardiothoracic surgery operations. In this analysis, the Model 5A ORs were 1.04 (95% CI, 0.76–1.43) for >0 to <10 min, 1.30 (95% CI, 1.01–1.67) for 10 to <30 min, and 1.77 (95% CI, 1.35–2.32) for ≥30 min. In the broad gastrointestinal procedure cohort, the corresponding ORs were 1.09 (95% CI, 0.80–1.48), 1.37 (95% CI, 1.07–1.75), and 1.98 (95% CI, 1.53–2.56). In the site-compatible strict cohort, the estimates remained directionally consistent; the ORs were 1.03 (95% CI, 0.74–1.44), 1.27 (95% CI, 0.97–1.66), and 1.68 (95% CI, 1.26–2.24), respectively. Thus, the association for ≥30 min remained statistically significant across all alternative cohort definitions, whereas the 10 to <30-min estimate did not reach statistical significance in the most restrictive site-compatible cohort.

### Missing data and multiple imputation

Of the 7,405 patients, 6,045 (81.6%) had complete data for the Model 3 covariates and 1,360 were excluded from the complete-case analyses. Missingness was highest for glucose (13.2%), albumin (11.8%), hemoglobin (11.5%), creatinine (10.0%), and BMI (1.0%). Complete cases differed from excluded patients in several clinical and procedural characteristics, although ICU admission rates were not significantly different (16.0% vs. 14.0%; *p* = 0.083). The prevalence of MAP <65 mmHg for at least 10 min was 55.4% among complete cases and 52.4% among excluded patients (*p* = 0.046).

Multiple imputation using 20 datasets produced results consistent with the complete-case expanded model. In the imputed Model 5A, the ORs were 1.10 (95% CI, 0.83–1.44; *p* = 0.518) for >0 to <10 min, 1.29 (95% CI, 1.04–1.62; *p* = 0.022) for 10 to <30 min, and 1.67 (95% CI, 1.31–2.12; *p* < 0.001) for ≥30 min.

### Subgroup analyses

**Figure 3:**
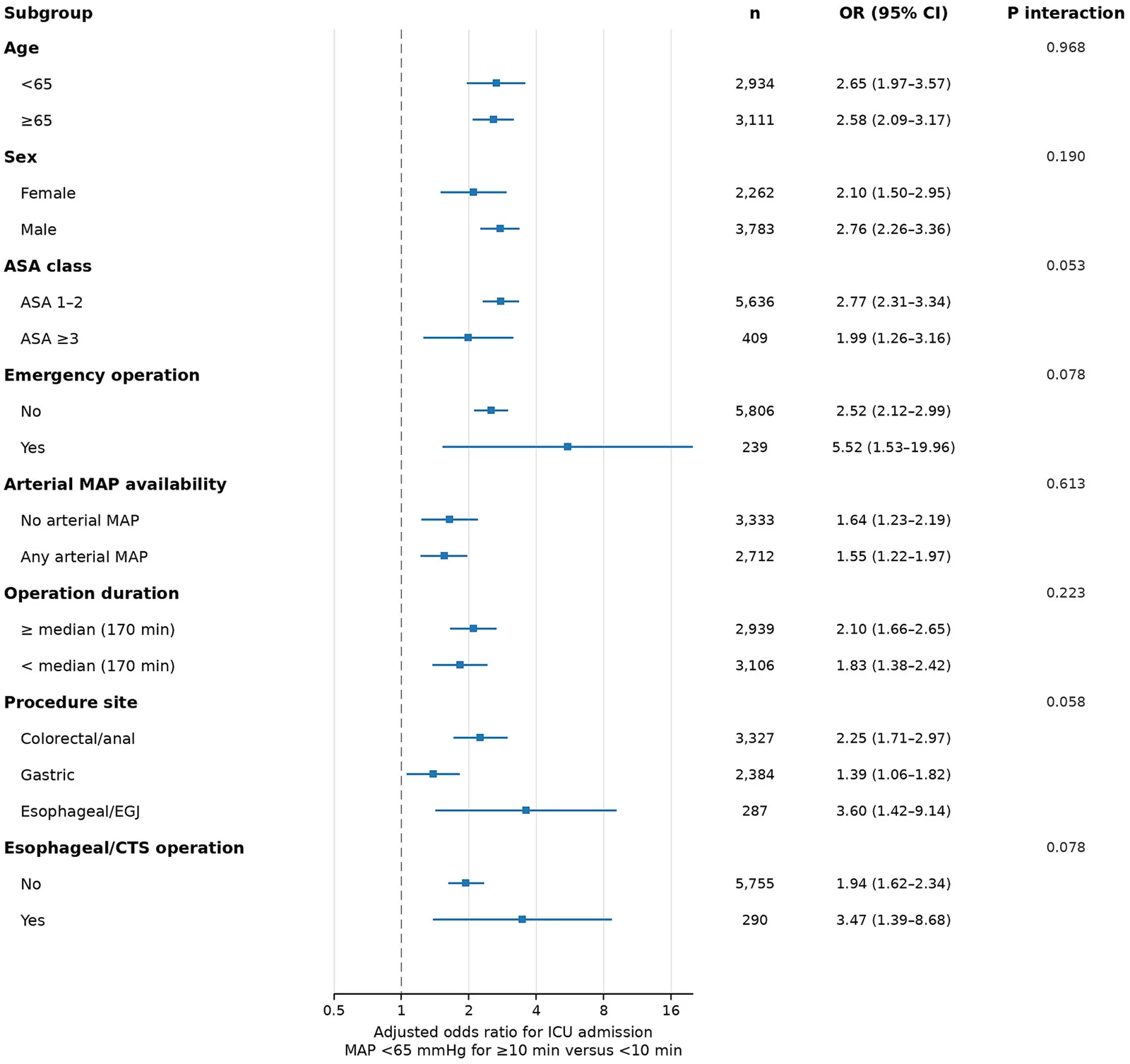
Subgroup analyses of MAP <65 mmHg for ≥10 min and postoperative ICU admission. Squares indicate adjusted odds ratios and horizontal lines indicate 95% confidence intervals. No significant interaction was identified across the prespecified subgroups. CI, confidence interval; ICU, intensive care unit; MAP, mean arterial pressure; OR, odds ratio.

MAP <65 mmHg for at least 10 min was associated with higher odds of ICU admission in the estimable age, sex, ASA, emergency-status, arterial-MAP-availability, operation-duration, and major procedure-site strata. However, no statistically significant effect modification was identified by age (*p* interaction = 0.968), sex (*p* interaction = 0.190), ASA class (*p* interaction = 0.053), emergency status (*p* interaction = 0.078), arterial MAP availability (*p* interaction = 0.613), operation duration (*p* interaction = 0.223), procedure site (*p* interaction = 0.058), or esophageal/cardiothoracic surgery status (*p* interaction = 0.078; Figure 3).

## Discussion

In this retrospective cohort study of 7,405 adults undergoing gastrointestinal lumen cancer excision or resection, longer cumulative exposure to intraoperative mean arterial pressure (MAP) below 65 mmHg was associated with a higher likelihood of postoperative intensive care unit (ICU) admission. The unadjusted and clinically adjusted analyses showed a strong graded relationship across increasing hypotension-duration categories. However, the magnitude of the association was substantially attenuated after accounting for operation duration, procedure site, surgical approach, and anesthesia type. In the expanded Model 5A, MAP <65 mmHg lasting <10 min was not associated with ICU admission, whereas exposure lasting 10 to <30 min was associated with a modest increase in odds and exposure lasting ≥30 min remained more clearly associated with ICU admission. Importantly, the findings remained consistent when hypotension was quantified as a time-weighted area integrating depth and duration and when burden was normalized to the monitored, strict operation, and strict anesthesia windows. These results indicate that part of the crude association reflects surgical duration and complexity, while prolonged hypotension burden retains prognostic information after adjustment for measured clinical and surgical factors.

The present findings are broadly consistent with previous work linking intraoperative hypotension to postoperative adverse outcomes. Large observational studies have identified MAP <65 mmHg as a clinically relevant threshold associated with acute kidney injury, myocardial injury after noncardiac surgery, and mortality, with risk increasing as the duration and severity of hypotension increase (8, 23). Systematic reviews and randomized evidence have also supported the general concept that avoiding sustained intraoperative hypotension may reduce postoperative morbidity in selected populations (5, 10, 12). Our study extends this literature by examining postoperative ICU admission in a specifically defined gastrointestinal lumen cancer surgical cohort and by reconstructing cumulative MAP exposure from high-density intraoperative time-series data. Importantly, the revised analysis also demonstrates that estimates based only on patient-level clinical adjustment can overstate the association when operation duration and procedure characteristics are not considered. This is consistent with a recent population-based analysis of complex gastrointestinal cancer surgery showing that care-delivery factors such as anesthesiologist procedural volume are independently associated with unplanned postoperative ICU admission, reinforcing that ICU utilization in this surgical population reflects a composite of physiological exposure and system-level practice rather than intraoperative hemodynamics alone (24).

The marked attenuation from Model 3 to Model 5A is clinically and methodologically important. In the clinical model, the adjusted odds ratios for the >0 to <10-min, 10 to <30-min, and ≥30-min categories were 1.43, 2.05, and 4.88, respectively. After surgical factors were added, the corresponding estimates were 1.02, 1.29, and 1.78. Longer and more complex procedures provide more opportunity for hypotension to accumulate and are also more likely to be followed by planned postoperative ICU monitoring. This concern was directly examined in the new time-normalized analyses. When MAP exposure was clipped to the operation-start to operation-end interval, each 5-min/h increase in MAP <65 mmHg duration remained associated with 14% higher odds of ICU admission, and the operation-window area integrating depth and duration showed a similar graded association. Estimates were stable when analyses were restricted to operations with at least 80% or 90% MAP coverage. These findings reduce the likelihood that the observed association is solely a mathematical consequence of longer procedures, although residual confounding by unmeasured operative complexity remains possible.

The results from esophageal and cardiothoracic procedures further illustrate this issue. Patients undergoing these operations had substantially longer procedures, greater hypotension burden, and very high ICU admission rates, consistent with routine or planned postoperative critical-care monitoring at the study institution. This pattern is consistent with recent esophagectomy cohorts in which unplanned ICU admission was independently associated with more extensive surgical exposure, including open or hybrid approach, longer operative time, and intraoperative transfusion, rather than occurring as a uniform institutional default (25). A related analysis further found that roughly half of critical complications after esophagectomy emerged only after postoperative day 5, arguing that the relationship between intraoperative physiology and ICU need extends well beyond the immediate perioperative window and is not fully captured by admission-day status alone (26). Excluding esophageal procedures and cardiothoracic operations did not materially alter the expanded-model estimates: MAP <65 mmHg for 10 to <30 min and ≥30 min remained associated with higher odds of ICU admission, whereas exposure lasting <10 min remained unassociated. This analysis reduces concern that the overall findings were driven entirely by a subgroup in which ICU admission was nearly universal, although it cannot eliminate confounding by other unmeasured aspects of operative complexity or local postoperative pathways.

The dose–response analyses also require a nuanced interpretation. ICU admission rates increased from 7.15% among patients with no MAP <65 mmHg exposure to 32.01% among those with ≥30 min of exposure. The overall spline association remained statistically significant after adjustment for surgical factors, but evidence of nonlinearity was no longer present in Models 4 and 5A. Thus, the revised results support a graded residual association rather than a sharply defined biological threshold or a distinctly nonlinear risk curve. The 10-min and 30-min categories are clinically interpretable exposure groupings, but they should not be considered validated intervention thresholds. In particular, the 10 to <30-min estimate was modest and did not reach statistical significance in the most restrictive site-compatible cohort, whereas the ≥30-min association remained statistically significant across all alternative cohort definitions.

Sensitivity analyses using alternative hypotension definitions reinforced the primary interpretation. Threshold-based, continuous-duration, and minimum-MAP measures were each associated with ICU admission after surgical adjustment. The time-weighted area below MAP 65 mmHg provided complementary evidence because it incorporated both hypotension depth and duration. Associations also remained when burden was expressed per monitored hour and when duration and area were recomputed strictly within the operation and anesthesia windows. The strict operation-window spline analyses supported graded associations without clear evidence of nonlinearity, and coverage-restricted analyses yielded similar estimates. Results were also consistent in the broad gastrointestinal procedure cohort, after substitution of cancer site for procedure site, after exclusion of esophageal or cardiothoracic operations, and after multiple imputation using 20 datasets (27). Together, these analyses reduce the likelihood that the findings were caused by a particular exposure definition, longer monitoring opportunity, incomplete operation-window coverage, the complete-case restriction, or one specific cohort definition. The variation in statistical significance for the 10 to <30-min category across the most restrictive cohort analyses nevertheless supports cautious emphasis on the more prolonged ≥30-min exposure group.

The biological plausibility of a residual association between sustained hypotension and postoperative care needs is supported by established principles of organ perfusion. Prolonged reductions in MAP may compromise oxygen delivery when autoregulatory reserve is exceeded, particularly in patients with anemia, hypoalbuminemia, malnutrition, systemic inflammation, or limited cardiopulmonary reserve (13, 17). Contemporary reviews of blood-pressure management in critically ill and perioperative patients similarly emphasize that adequate perfusion pressure, rather than a single absolute MAP value, determines end-organ oxygen delivery, and that patients with reduced physiological reserve are most vulnerable to harm once autoregulatory limits are exceeded (28). These mechanisms can contribute to organ dysfunction and a greater need for postoperative monitoring or support. However, the present data cannot determine whether hypotension directly caused the subsequent ICU admission. Hypotension may instead arise from bleeding, vasodilation, cardiac dysfunction, difficult dissection, or other intraoperative events that themselves influence postoperative disposition. The residual association should therefore be interpreted as prognostic rather than causal.

Postoperative ICU admission is a clinically important but heterogeneous outcome. It reflects resource use and escalation of care, but it is not equivalent to a postoperative complication. ICU admission may be planned before surgery because of the anticipated complexity of the procedure, the patient’s baseline risk, the need for invasive monitoring, or institutional care pathways. It may also be influenced by bed availability and clinician preference. The INSPIRE database did not contain a validated indicator distinguishing planned from unplanned ICU admission. This limitation is not unique to the present cohort: dedicated analyses of esophagectomy patients have shown that unplanned and planned ICU admissions carry different risk-factor profiles and different downstream mortality and resource implications, underscoring that any single “ICU admission” outcome variable conflates distinct clinical processes (25, 26). Consequently, the outcome in this study should be understood as a mixed marker of postoperative monitoring requirements, perioperative risk, surgical complexity, and institutional practice. This limitation is particularly relevant to esophageal and cardiothoracic procedures and explains why the findings should not be interpreted as evidence that hypotension necessarily caused clinical deterioration.

No statistically significant effect modification was identified across the prespecified subgroups. The association between MAP <65 mmHg for at least 10 min and ICU admission was directionally similar across age, sex, ASA class, emergency status, invasive arterial MAP availability, operation duration, procedure site, and esophageal/cardiothoracic surgery status. Several interaction *p* values were close to 0.05, including those for ASA class and procedure site, but these findings should be considered exploratory because multiple interaction tests were performed and some subgroup strata were relatively small. Overall, the subgroup analyses support broad consistency rather than clear evidence for a population in which the association is substantially stronger or weaker.

### Strengths and limitations

This study has several strengths. The cohort was reconstructed using procedure-specific ICD-10-PCS codes combined with temporally aligned cancer diagnoses, improving the specificity of case identification. Hypotension was evaluated using complementary duration, minimum-MAP, time-weighted area, and time-normalized measures. Strict operation- and anesthesia-window analyses showed high MAP coverage, and results remained consistent across multiple imputation, alternative cohort definitions, coverage-restricted analyses, and exclusion of esophageal/cardiothoracic procedures. Expanded models also adjusted for major surgical characteristics without evidence of problematic multicollinearity.

Several limitations should be acknowledged. The retrospective observational design precludes causal inference, and residual confounding may remain because cancer stage, detailed comorbidities, blood loss, transfusion, fluid therapy, and vasopressor exposure were not fully available. Planned and unplanned ICU admissions could not be distinguished, limiting outcome specificity. MAP values were predominantly recorded at 5-min intervals, so brief fluctuations between measurements may have been missed. Concurrent procedures could not be comprehensively identified from the shortened procedure codes. Missing covariate data required complete-case analysis, although multiple imputation produced similar results. Finally, the single-center South Korean setting may limit generalizability.

### Clinical implications and future research

The findings have potential implications for perioperative risk assessment but should not be used to define a treatment threshold. Brief MAP <65 mmHg exposure lasting <10 min was not associated with ICU admission after adjustment for surgical factors, whereas more sustained exposure was associated with progressively higher odds. Prolonged hypotension, especially cumulative exposure of ≥30 min, may therefore identify patients with greater perioperative risk or more complex intraoperative courses who could benefit from closer postoperative assessment. However, the present study does not establish that preventing hypotension will reduce ICU admission, nor does it show that ICU admission itself represents an avoidable adverse event.

External validation is needed in databases that can distinguish planned from unplanned ICU admission and provide detailed information on comorbidities, cancer stage, bleeding, transfusion, fluid therapy, vasoactive treatment, and postoperative complications. Future studies should also evaluate organ-specific outcomes, mortality, and unplanned escalation of care. Prospective interventional studies are required to determine whether individualized blood-pressure targets, earlier treatment of sustained hypotension, or protocolized hemodynamic management can improve patient-centered outcomes in gastrointestinal cancer surgery. Such studies should account for baseline blood pressure and patient-specific autoregulatory reserve rather than relying exclusively on a single absolute MAP threshold.

## Conclusion

Among adults undergoing gastrointestinal lumen cancer excision or resection, cumulative intraoperative MAP <65 mmHg duration was associated with postoperative ICU admission after adjustment for measured clinical and surgical factors. Exposure lasting <10 min was not associated with ICU admission in the expanded model, while the 10 to <30-min category showed a modest association and the ≥30-min category demonstrated the most consistent association across sensitivity analyses. Complementary analyses using time-weighted area and strict operation- and anesthesia-window normalization supported a graded association that was not explained solely by longer procedures or monitoring time. These findings identify prolonged hypotension burden as a marker of postoperative care needs and perioperative risk, but do not establish causality or a treatment threshold.

## Data Availability

The original contributions presented in the study are included in the article/supplementary material, further inquiries can be directed to the corresponding author/s.
