# Supplementary material for: Association between cumulative intraoperative hypotension and postoperative ICU admission after gastrointestinal lumen cancer surgery: a retrospective cohort study using the INSPIRE database
Source: Front Med (Lausanne). 2026 Aug 19;13:1916589. doi: 10.3389/fmed.2026.1916589 (PMC13534046; doi:10.3389/fmed.2026.1916589)
Supplement: Supplementary file 1 [file Supplementary_file_1.docx]

**Supplementary Material**

**Association Between Cumulative Intraoperative Hypotension and Postoperative ICU Admission After Gastrointestinal Lumen Cancer Surgery: A Retrospective Cohort Study Using the INSPIRE Database**

# Supplementary methods

MAP measurement intervals and time-weighted area. Invasive arterial MAP was preferred when invasive and noninvasive MAP were recorded at the same timestamp. Values outside 20–250 mmHg were excluded. Consecutive MAP intervals were summarized empirically. Each MAP value represented the interval until the next valid value, capped at 5 min. The time-weighted area below MAP 65 mmHg was calculated as the sum of the deficit below 65 mmHg multiplied by the represented interval; the area below MAP 60 mmHg was calculated analogously. Areas were expressed in mmHg·min.

Time-normalized and strict-window metrics. Whole-window metrics included the percentage of represented MAP time below 65 mmHg and minutes below 65 mmHg per monitored hour. For strict operation and anesthesia analyses, each represented MAP interval was clipped to its overlap with the recorded operation-start/operation-end or anesthesia-start/anesthesia-end window. Duration and area were then normalized per hour of the corresponding window. MAP coverage was calculated as represented MAP time within the window divided by window duration.

Statistical analyses. New time-weighted and normalized exposures were evaluated using Model 5A, adjusted for age, sex, ASA class, emergency operation, BMI, hemoglobin, albumin, creatinine, glucose, operation duration, procedure site, surgical approach, and anesthesia type. Effects were reported per 50 mmHg·min for whole-window area, per 5 percentage points of monitored time, per 5 min/h of normalized duration, and per 25 mmHg·min/h of normalized area. Strict operation-window analyses were repeated among operations with MAP coverage ≥80% and ≥90%. Restricted cubic splines with 4 degrees of freedom evaluated the overall and nonlinear associations for strict operation-window duration and area.

# Supplementary results

Multiple-imputation analyses, alternative surgical adjustment using cancer site, and analyses based on alternative cohort definitions were consistent with the principal complete-case findings. After adjustment for measured clinical and surgical factors, exposure lasting less than 10 min was not associated with postoperative ICU admission, whereas the association was most consistent for cumulative MAP <65 mmHg lasting at least 30 min. The estimate for 10–<30 min was modest and did not reach statistical significance in the most restrictive site-compatible cohort.

The corrected collinearity assessment showed maximum variance inflation factors below 2.0 in both expanded models. Complete cases and excluded patients differed in several clinical and procedural characteristics; however, postoperative ICU admission rates were not statistically different. Multiple imputation using 20 datasets was therefore used to evaluate the sensitivity of the complete-case estimates to missing BMI and laboratory values.

## MAP measurement intervals and window coverage

The cohort contributed 327,066 selected MAP records and 319,661 consecutive intervals. The median interval was 5 min (IQR, 5–5); 99.43% of intervals were exactly 5 min, 0.44% were >5–≤10 min, and 0.12% were >10 min. The strict operation window was valid for all 7,405 operations. Median operation-window MAP coverage was 100% (IQR, 98.85%–100%), with 98.76% and 95.76% of operations meeting coverage thresholds of ≥80% and ≥90%, respectively. The strict anesthesia window was valid for 7,392 operations and had median coverage of 98.32% (IQR, 96.41%–100%) (Supplementary Tables S7 and S8).

## Time-weighted and normalized hypotension burden

The median area below MAP 65 mmHg across the whole monitoring window was 32.5 mmHg·min (IQR, 0–100), and 70.93% of patients had a nonzero area. Within the strict operation window, the median MAP <65 mmHg duration was 5 min (IQR, 0–15), the median area was 15 mmHg·min (IQR, 0–60), and the median normalized duration was 1.71 min/h (IQR, 0–5.46) (Supplementary Table S9).

In Model 5A, the whole-window area below MAP 65 mmHg was associated with ICU admission (OR per 50 mmHg·min, 1.07; 95% CI, 1.04–1.10; P < 0.001). Each 5-min/h increase in whole-window duration per monitored hour was associated with an OR of 1.15 (95% CI, 1.08–1.23; P < 0.001). Within the strict operation window, the corresponding ORs were 1.14 (95% CI, 1.07–1.21) per 5 min/h of duration and 1.11 (95% CI, 1.06–1.17) per 25 mmHg·min/h of area (both P < 0.001). Strict anesthesia-window estimates were similar (Supplementary Table S10).

Operation-window estimates remained stable when analyses were restricted to MAP coverage ≥80% or ≥90%. Restricted cubic spline analyses showed significant overall associations for normalized operation-window duration and area (both P < 0.001), without clear evidence of nonlinearity (P = 0.174 and P = 0.377, respectively) (Supplementary Table S11).

**Supplementary Table S1. Multiple-imputation analyses of cumulative MAP <65 mmHg duration and postoperative ICU admission**

| **Model** | **n** | **ICU events** | **>0–<10 min** | **10–<30 min** | **≥30 min** |
| --- | --- | --- | --- | --- | --- |
| MI Model 3 | 7,405 | 1,157 | 1.40 (1.08–1.81); P = 0.010 | 1.89 (1.54–2.32); P < 0.001 | 4.14 (3.36–5.12); P < 0.001 |
| MI Model 4 | 7,405 | 1,157 | 1.16 (0.88–1.52); P = 0.294 | 1.33 (1.07–1.65); P = 0.011 | 1.63 (1.29–2.06); P < 0.001 |
| MI Model 5A | 7,405 | 1,157 | 1.10 (0.83–1.44); P = 0.518 | 1.29 (1.04–1.62); P = 0.022 | 1.67 (1.31–2.12); P < 0.001 |

*Note.* Values are pooled odds ratios (95% confidence intervals) with 0 min as the reference category. Missing BMI, hemoglobin, albumin, creatinine, and glucose values were imputed using chained equations with Bayesian ridge regression and posterior sampling across 20 imputed datasets; estimates were combined using Rubin's rules. Model 3 adjusted for age, sex, ASA class, emergency operation, BMI, hemoglobin, albumin, creatinine, and glucose. Model 4 additionally adjusted for observed MAP interval. Model 5A replaced the monitoring-time adjustment with operation duration, procedure site, surgical approach, and anesthesia type. ASA, American Society of Anesthesiologists; ICU, intensive care unit; MAP, mean arterial pressure; MI, multiple imputation.

**Supplementary Table S2. Alternative expanded adjustment using cancer site instead of procedure site**

| **Model** | **n** | **ICU events** | **>0–<10 min** | **10–<30 min** | **≥30 min** |
| --- | --- | --- | --- | --- | --- |
| Model 5A: procedure site | 6,045 | 966 | 1.02 (0.75–1.40); P = 0.880 | 1.29 (1.00–1.66); P = 0.047 | 1.78 (1.36–2.33); P < 0.001 |
| Model 5B: cancer site | 6,045 | 966 | 1.06 (0.77–1.45); P = 0.717 | 1.30 (1.01–1.68); P = 0.038 | 1.79 (1.36–2.34); P < 0.001 |

*Note.* Values are adjusted odds ratios (95% confidence intervals) with 0 min as the reference category. Both models adjusted for age, sex, ASA class, emergency operation, BMI, hemoglobin, albumin, creatinine, glucose, operation duration, surgical approach, and anesthesia type. Model 5A included procedure site; Model 5B replaced procedure site with cancer site. Procedure site and cancer site were not entered simultaneously.

**Supplementary Table S3. Sensitivity analyses using alternative cohort definitions**

| **Analysis cohort** | **Complete-case n** | **ICU events** | **>0–<10 min** | **10–<30 min** | **≥30 min** |
| --- | --- | --- | --- | --- | --- |
| Primary strict cohort | 6,045 | 966 | 1.02 (0.75–1.40); P = 0.880 | 1.29 (1.00–1.66); P = 0.047 | 1.78 (1.36–2.33); P < 0.001 |
| Excluding esophageal/CTS operations | 5,755 | 730 | 1.04 (0.76–1.43); P = 0.802 | 1.30 (1.01–1.67); P = 0.042 | 1.77 (1.35–2.32); P < 0.001 |
| Broad GI procedure cohort | 6,371 | 1,044 | 1.09 (0.80–1.48); P = 0.575 | 1.37 (1.07–1.75); P = 0.011 | 1.98 (1.53–2.56); P < 0.001 |
| Site-compatible strict cohort | 5,137 | 874 | 1.03 (0.74–1.44); P = 0.866 | 1.27 (0.97–1.66); P = 0.085 | 1.68 (1.26–2.24); P < 0.001 |

*Note.* Values are Model 5A odds ratios (95% confidence intervals), with 0 min as the reference category. The primary, esophageal/CTS-excluded, and broad-cohort models adjusted for age, sex, ASA class, emergency operation, BMI, hemoglobin, albumin, creatinine, glucose, operation duration, procedure site, surgical approach, and anesthesia type. In the site-compatible cohort, sparse procedure categories were collapsed, non-open approaches were combined, and anesthesia type was omitted because the non-general category was sparse. CTS, cardiothoracic surgery; GI, gastrointestinal; ICU, intensive care unit; MAP, mean arterial pressure.

**Supplementary Table S4. Corrected variance inflation factor assessment for the expanded models**

| **Model** | **Complete-case n** | **Design-matrix columns, including intercept** | **Maximum VIF** | **Interpretation** |
| --- | --- | --- | --- | --- |
| Model 5A: clinical + surgical factors | 6,045 | 19 | 1.82 | No concerning multicollinearity |
| Model 5B: clinical + cancer site | 6,045 | 19 | 1.90 | No concerning multicollinearity |

*Note.* VIFs were calculated from treatment-coded design matrices that included an intercept and matched the parameterization of the logistic regression models. VIF, variance inflation factor.

**Supplementary Table S5. Missingness of variables included in the complete-case clinical and expanded models**

| **Variable** | **Missing, n** | **Missing, %** | **Available, n** |
| --- | --- | --- | --- |
| BMI | 76 | 1.0 | 7,329 |
| Hemoglobin | 848 | 11.5 | 6,557 |
| Albumin | 875 | 11.8 | 6,530 |
| Creatinine | 742 | 10.0 | 6,663 |
| Glucose | 975 | 13.2 | 6,430 |

*Note.* The analytic cohort contained 7,405 patients. The outcome, hypotension exposure, age, sex, ASA class, emergency status, operation duration, observed MAP interval, procedure site, surgical approach, anesthesia type, and cancer site had no missing values. Variable-specific missingness overlapped; therefore, missing counts do not sum to the 1,360 patients excluded from complete-case models. BMI, body mass index; MAP, mean arterial pressure.

**Supplementary Table S6. Comparison of complete cases and patients excluded because of missing covariates**

| **Variable** | **Level/statistic** | **All patients (n = 7,405)** | **Complete cases (n = 6,045)** | **Excluded (n = 1,360)** | **P value** |
| --- | --- | --- | --- | --- | --- |
| Age, years | Median (IQR) | 65.00 (55.00, 70.00) | 65.00 (55.00, 70.00) | 60.00 (55.00, 70.00) | <0.001 |
| BMI, kg/m2 | Median (IQR) | 22.89 (20.76, 25.39) | 22.89 (20.76, 25.39) | 22.89 (20.76, 25.39) | 0.650 |
| Hemoglobin, g/dL | Median (IQR) | 12.90 (11.60, 14.20) | 13.20 (11.60, 14.20) | 12.50 (11.30, 13.70) | <0.001 |
| Albumin, g/dL | Median (IQR) | 4.00 (3.80, 4.30) | 4.00 (3.80, 4.30) | 3.90 (3.60, 4.10) | <0.001 |
| Creatinine, mg/dL | Median (IQR) | 0.84 (0.71, 0.98) | 0.80 (0.68, 0.98) | 0.84 (0.71, 0.98) | 0.052 |
| Glucose, mg/dL | Median (IQR) | 101.00 (91.00, 120.00) | 101.00 (91.00, 115.00) | 105.00 (91.00, 126.00) | 0.004 |
| Operation duration, min | Median (IQR) | 170.0 (105.0, 225.0) | 165.0 (105.0, 225.0) | 185.0 (125.0, 230.0) | <0.001 |
| Observed MAP interval, min | Median (IQR) | 205.0 (140.0, 265.0) | 205.0 (140.0, 265.0) | 220.0 (160.0, 270.0) | <0.001 |
| MAP <65 duration, min | Median (IQR) | 10.0 (0.0, 25.0) | 10.0 (0.0, 25.0) | 10.0 (0.0, 20.0) | 0.020 |
| ASA class | 1 | 2,353 (31.8%) | 1,856 (30.7%) | 497 (36.5%) | <0.001 |
| ASA class | 2 | 4,572 (61.7%) | 3,780 (62.5%) | 792 (58.2%) |  |
| ASA class | ≥3 | 480 (6.5%) | 409 (6.8%) | 71 (5.2%) |  |
| Emergency operation | No | 7,094 (95.8%) | 5,806 (96.0%) | 1,288 (94.7%) | 0.031 |
| Emergency operation | Yes | 311 (4.2%) | 239 (4.0%) | 72 (5.3%) |  |
| MAP <65 mmHg for ≥10 min | No | 3,345 (45.2%) | 2,697 (44.6%) | 648 (47.6%) | 0.046 |
| MAP <65 mmHg for ≥10 min | Yes | 4,060 (54.8%) | 3,348 (55.4%) | 712 (52.4%) |  |
| Postoperative ICU admission | No | 6,248 (84.4%) | 5,079 (84.0%) | 1,169 (86.0%) | 0.083 |
| Postoperative ICU admission | Yes | 1,157 (15.6%) | 966 (16.0%) | 191 (14.0%) |  |
| Procedure site | Colorectal/anal | 3,777 (51.0%) | 3,327 (55.0%) | 450 (33.1%) | <0.001 |
| Procedure site | Gastric | 3,236 (43.7%) | 2,384 (39.4%) | 852 (62.6%) |  |
| Procedure site | Small bowel/appendix | 46 (0.6%) | 37 (0.6%) | 9 (0.7%) |  |
| Procedure site | Esophageal/EGJ | 334 (4.5%) | 287 (4.7%) | 47 (3.5%) |  |
| Procedure site | Other/unspecified | 12 (0.2%) | 10 (0.2%) | 2 (0.1%) |  |

*Note.* Continuous variables are presented as median (IQR), and categorical variables as n (%). P values were calculated using the Mann-Whitney U test for continuous variables and the chi-square test for categorical variables. Complete cases had nonmissing age, sex, ASA class, emergency status, BMI, hemoglobin, albumin, creatinine, and glucose. ASA, American Society of Anesthesiologists; BMI, body mass index; EGJ, esophagogastric junction; ICU, intensive care unit; IQR, interquartile range; MAP, mean arterial pressure.

**Supplementary Table S7. Distribution of consecutive MAP measurement intervals**

| **Interval source** | **n** | **P25, min** | **Median, min** | **P75, min** | **P95, min** | **P99, min** | **Maximum, min** |
| --- | --- | --- | --- | --- | --- | --- | --- |
| All selected MAP intervals | 319,661 | 5.0 | 5.0 | 5.0 | 5.0 | 5.0 | 1,420.0 |
| Intervals starting with arterial MAP | 159,143 | 5.0 | 5.0 | 5.0 | 5.0 | 5.0 | 575.0 |
| Intervals starting with noninvasive MAP | 160,518 | 5.0 | 5.0 | 5.0 | 5.0 | 5.0 | 1,420.0 |
| Operation-specific median interval | 7,405 | 5.0 | 5.0 | 5.0 | 5.0 | 5.0 | 35.0 |

Note. Intervals were calculated before applying the 5-min cap used for exposure construction. MAP, mean arterial pressure.

**Supplementary Table S8. MAP interval categories and coverage of strict operation and anesthesia windows**

| **Panel** | **Measure** | **Category or statistic** | **n** | **Denominator** | **Percentage or value** |
| --- | --- | --- | --- | --- | --- |
| A | Consecutive MAP interval | ≤1 min | 0 | 319,661 | 0.00% |
| A | Consecutive MAP interval | >1–<5 min | 0 | 319,661 | 0.00% |
| A | Consecutive MAP interval | 5 min | 317,853 | 319,661 | 99.43% |
| A | Consecutive MAP interval | >5–≤10 min | 1,418 | 319,661 | 0.44% |
| A | Consecutive MAP interval | >10 min | 390 | 319,661 | 0.12% |
| B | Operation-window MAP coverage | Median (IQR) | 7,405 | 7,405 | 100.00% (98.85%–100.00%) |
| B | Operation-window MAP coverage | ≥80% | 7,313 | 7,405 | 98.76% |
| B | Operation-window MAP coverage | ≥90% | 7,091 | 7,405 | 95.76% |
| C | Anesthesia-window MAP coverage | Median (IQR) | 7,392 | 7,392 | 98.32% (96.41%–100.00%) |
| C | Anesthesia-window MAP coverage | ≥80% | 7,336 | 7,392 | 99.24% |
| C | Anesthesia-window MAP coverage | ≥90% | 7,070 | 7,392 | 95.64% |

Note. Panel A summarizes raw consecutive intervals. Panels B and C summarize the percentage of each strict window covered by represented MAP intervals. Thirteen operations lacked a valid anesthesia window. IQR, interquartile range; MAP, mean arterial pressure.

**Supplementary Table S9. Distribution of time-weighted and normalized hypotension metrics**

| **Metric** | **n** | **P25** | **Median** | **P75** | **P95** | **P99** | **Maximum** | **Nonzero, %** |
| --- | --- | --- | --- | --- | --- | --- | --- | --- |
| Area below MAP 65, whole window, mmHg·min | 7,405 | 0.000 | 32.500 | 100.000 | 327.000 | 717.400 | 2,825.000 | 70.93 |
| Area below MAP 60, whole window, mmHg·min | 7,405 | 0.000 | 0.000 | 22.500 | 120.000 | 320.000 | 1,600.000 | 40.65 |
| MAP <65, % of monitored time | 7,405 | 0.000 | 4.762 | 11.538 | 28.571 | 46.958 | 88.889 | 70.93 |
| MAP <65 duration, strict operation window, min | 7,405 | 0.000 | 5.000 | 15.000 | 50.000 | 114.800 | 300.000 | 58.42 |
| Area below MAP 65, strict operation window, mmHg·min | 7,405 | 0.000 | 15.000 | 60.000 | 235.000 | 569.800 | 2,825.000 | 58.42 |
| MAP <65 duration per operation hour, min/h | 7,405 | 0.000 | 1.714 | 5.455 | 16.744 | 29.164 | 60.000 | 58.42 |
| Area below MAP 65 per operation hour, mmHg·min/h | 7,405 | 0.000 | 4.000 | 20.357 | 77.865 | 168.321 | 525.000 | 58.42 |
| MAP <65 duration per anesthesia hour, min/h | 7,392 | 0.000 | 2.791 | 6.885 | 16.957 | 27.416 | 54.286 | 70.75 |
| Area below MAP 65 per anesthesia hour, mmHg·min/h | 7,392 | 0.000 | 10.000 | 29.016 | 85.067 | 168.982 | 454.286 | 70.75 |

Note. Whole-window metrics used all capped represented intervals. Strict-window metrics included only interval overlap with the recorded operation or anesthesia window. MAP, mean arterial pressure.

**Supplementary Table S10. Model 5A associations for time-weighted and normalized hypotension metrics**

| **Exposure metric** | **Comparison or increment** | **n** | **ICU events** | **Adjusted OR (95% CI)** | **P value** |
| --- | --- | --- | --- | --- | --- |
| Area below MAP 65, whole monitoring window | Per 50-mmHg·min increase | 6,045 | 966 | 1.07 (1.04–1.10) | <0.001 |
| Area below MAP 60, whole monitoring window | Per 25-mmHg·min increase | 6,045 | 966 | 1.08 (1.05–1.12) | <0.001 |
| MAP <65, percentage of monitored time | Per 5-percentage-point increase | 6,045 | 966 | 1.09 (1.05–1.13) | <0.001 |
| MAP <65 duration per monitored hour | Per 5-min/h increase | 6,045 | 966 | 1.15 (1.08–1.23) | <0.001 |
| MAP <65 duration per operation hour, strict window | Per 5-min/h increase | 6,045 | 966 | 1.14 (1.07–1.21) | <0.001 |
| Area below MAP 65 per operation hour, strict window | Per 25-mmHg·min/h increase | 6,045 | 966 | 1.11 (1.06–1.17) | <0.001 |
| MAP <65 duration per anesthesia hour, strict window | Per 5-min/h increase | 6,034 | 965 | 1.15 (1.07–1.23) | <0.001 |
| Area below MAP 65 per anesthesia hour, strict window | Per 25-mmHg·min/h increase | 6,034 | 965 | 1.13 (1.07–1.19) | <0.001 |

Note. All estimates were adjusted for age, sex, ASA class, emergency operation, BMI, hemoglobin, albumin, creatinine, glucose, operation duration, procedure site, surgical approach, and anesthesia type. AUC, area under the curve; ICU, intensive care unit; MAP, mean arterial pressure; OR, odds ratio.

**Supplementary Table S11. Coverage-restricted and spline analyses of strict operation-window hypotension burden**

| **Analysis** | **Exposure metric** | **Increment** | **n** | **ICU events** | **OR (95% CI) or P value** | **P value / nonlinearity** |
| --- | --- | --- | --- | --- | --- | --- |
| Coverage ≥80% | MAP <65, % of operation time | Per 5 percentage points | 5,970 | 961 | 1.08 (1.04–1.12) | <0.001 |
| Coverage ≥80% | MAP <65 duration per operation hour | Per 5 min/h | 5,970 | 961 | 1.14 (1.07–1.21) | <0.001 |
| Coverage ≥80% | Area below MAP 65 per operation hour | Per 25 mmHg·min/h | 5,970 | 961 | 1.12 (1.06–1.18) | <0.001 |
| Coverage ≥90% | MAP <65, % of operation time | Per 5 percentage points | 5,782 | 944 | 1.08 (1.04–1.12) | <0.001 |
| Coverage ≥90% | MAP <65 duration per operation hour | Per 5 min/h | 5,782 | 944 | 1.13 (1.06–1.20) | <0.001 |
| Coverage ≥90% | Area below MAP 65 per operation hour | Per 25 mmHg·min/h | 5,782 | 944 | 1.11 (1.05–1.17) | <0.001 |
| Restricted cubic spline | MAP <65 duration per operation hour | 4 df | 6,045 | 966 | P overall < 0.001 | P nonlinear = 0.174 |
| Restricted cubic spline | Area below MAP 65 per operation hour | 4 df | 6,045 | 966 | P overall < 0.001 | P nonlinear = 0.377 |

Note. Coverage-restricted estimates used Model 5A. Restricted cubic spline tests compared reduced, linear, and spline models. df, degrees of freedom; ICU, intensive care unit; MAP, mean arterial pressure; OR, odds ratio.
